# Exploring the pathogenetic association between schizophrenia and type 2 diabetes mellitus diseases based on pathway analysis

**DOI:** 10.1186/1755-8794-6-S1-S17

**Published:** 2013-01-23

**Authors:** Yanli Liu, Zezhi Li, Meixia Zhang, Youping Deng, Zhenghui Yi, Tieliu Shi

**Affiliations:** 1 Center for Bioinformatics and Computational Biology, and The Institute of Biomedical Sciences, School of Life Sciences, East China Normal University, 500 Dongchuan Road, Shanghai 200241, China; 2 Department of Neurology, Shanghai Changhai Hospital, Secondary Military Medical University, 168 Changhai Road, Shanghai, China; 3 Department of Ophthalmology, West China Hospital, Sichuan University 37 Guoxuexiang, Chengdu, Sichuan, 610041, China; 4 Rush University Cancer Center, Department of Internal Medicine, Rush University Medical Center, Chicago, IL 60612, USA; 5 Schizophrenia Program, Shanghai Mental Health Center, Shanghai Jiao Tong University School of Medicine, 600 Wan Ping Nan Road, Shanghai 200030, China

## Abstract

**Background:**

Schizophrenia (SCZ) and type 2 diabetes mellitus (T2D) are both complex diseases. Accumulated studies indicate that schizophrenia patients are prone to present the type 2 diabetes symptoms, but the potential mechanisms behind their association remain unknown. Here we explored the pathogenetic association between SCZ and T2D based on pathway analysis and protein-protein interaction.

**Results:**

With sets of prioritized susceptibility genes for SCZ and T2D, we identified significant pathways (with adjusted p-value < 0.05) specific for SCZ or T2D and for both diseases based on pathway enrichment analysis. We also constructed a network to explore the crosstalk among those significant pathways. Our results revealed that some pathways are shared by both SCZ and T2D diseases through a number of susceptibility genes. With 382 unique susceptibility proteins for SCZ and T2D, we further built a protein-protein interaction network by extracting their nearest interacting neighbours. Among 2,104 retrieved proteins, 364 of them were found simultaneously interacted with susceptibility proteins of both SCZ and T2D, and proposed as new candidate risk factors for both diseases. Literature mining supported the potential association of partial new candidate proteins with both SCZ and T2D. Moreover, some proteins were hub proteins with high connectivity and interacted with multiple proteins involved in both diseases, implying their pleiotropic effects for the pathogenic association. Some of these hub proteins are the components of our identified enriched pathways, including calcium signaling, g-secretase mediated ErbB4 signaling, adipocytokine signaling, insulin signaling, AKT signaling and type II diabetes mellitus pathways. Through the integration of multiple lines of information, we proposed that those signaling pathways, which contain susceptibility genes for both diseases, could be the key pathways to bridge SCZ and T2D. AKT could be one of the important shared components and may play a pivotal role to link both of the pathogenetic processes.

**Conclusions:**

Our study is the first network and pathway-based systematic analysis for SCZ and T2D, and provides the general pathway-based view of pathogenetic association between two diseases. Moreover, we identified a set of candidate genes potentially contributing to the linkage between these two diseases. This research offers new insights into the potential mechanisms underlying the co-occurrence of SCZ and T2D, and thus, could facilitate the inference of novel hypotheses for the co-morbidity of the two diseases. Some etiological factors that exert pleiotropic effects shared by the significant pathways of two diseases may have important implications for the diseases and could be therapeutic intervention targets.

## Background

Schizophrenia (SCZ) is a chronic, severe, and disabling brain disorder that has affected people with lifelong disability. The phenotype is heterogeneous and complex, with multiple genes and environmental exposures likely involved. It is characterized by a breakdown of thought processes and by poor emotional responsiveness. It most commonly manifests itself as auditory hallucinations, paranoid or bizarre delusions, or disorganized speech and thinking, and it is accompanied by significant social or occupational dysfunction. The onset of symptoms typically occurs in young adulthood with 1% prevalence in the general population [1]. Recently, researchers have identified specific genes/markers and chromosomal regions for SCZ through numerous genetic studies, such as linkage scans and their meta-analyses, candidate gene association analyses, gene expression and genome-wide association studies (GWAS) [2-5].

Type 2 diabetes mellitus (T2D) is characterized by persistent high blood glucose in the context of insulin resistance and relative insulin deficiency, due to pancreatic beta-cell dysfunction. Cardiovascular diseases, chronic renal failure, retinal, and nerve damage are usual complications of this illness. Many genes and pathways have also been implicated with the T2D, but the mechanisms underlying the connections remain further investigation.

Recently studies indicate that the prevalence of T2D among individuals suffering from schizophrenia or schizoaffective disorders is significant higher than that of the general population [6,7]. For instance, a recent study reported that T2D is more common in schizophrenics than normal controls in Canada, especially in young males and females [8]. Another recent study also reported an elevated risk of T2D in schizophrenic individuals in Taiwan [9].

Molecular inference and GWAS studies also point out that SCZ shares substantial polygenetic component with T2D. Increased attention is now being given to a possible genetic basis for co-morbidity of SCZ and T2D [10]. The pathogenetic association between SCZ and T2D has been recognized but the potential mechanism behind the association has not been fully explored [10]. Recently, more and more researchers have paid their attentions to identify the candidate genes for human diseases, including T2D and SCZ, mainly through genome-wide association, transcriptomic and proteomic expression studies. These have greatly facilitated the research of genetic basis for pathogenetic association between SCZ and T2D. It is well accepted that genes or proteins usually interact with each other to form complexes or pathways within a cell, rather than function alone to carry out biological functions [11]. Considering that SCZ and T2D are both complex diseases, their pathogenesis is believed coupled with lots of factors. Lin has proposed three models for hypotheses concerning the co-morbidity between SCZ and T2D [10]. One of the models suggested that T2D and SCZ are caused by shared etiological factors, which is consistent with other research result that T2D and SCZ are caused by multiple genetic variants [12]. From this perspective, we can link these two diseases by their shared susceptibility genes. Those genes may exert pleiotropic effects; it means they play roles in two different pathological pathways, one related to SCZ and the other associated with T2D. For example, TCF7L2, one of the best confirmed susceptibility genes for T2D, has been also inferred to strongly relate to SCZ. On one hand, TCF7L2 acts a role in pancreatic beta cell function; on the other hand, it is a transcription factor involved in the Wnt/beta-catenin signaling [13]. Since Wnt signaling pathway plays a role in the development of central nervous system (CNS) [14], and has been also associated with SCZ [15,16], TCF7L2 may contribute to the co-morbidity of SCZ and T2D through Wnt signaling pathway [17]. In addition to genetic factors, environmental factors may also influence susceptibility to both SCZ and T2D, and anti-psychotic medications can also trigger the pathogenetic association between SCZ and T2D. Although significant attentions have been paid to explore the association between SCZ and T2D, not much progress has been made and the potential mechanisms remain unclear.

It is hypothesized that many genes may contribute major risk to SCZ through their interaction and combined effects, with each gene might contribute a small or moderate risk. Similarly, T2D has also been regarded as a complex disease and associated with the dysfunctions of multiple genes. Therefore, we assumed that proteins that interact with both SCZ proteins and T2D proteins should also be the potential ones to contribute to both diseases. Accordingly, in this study, we used those susceptibility genes that have been implicated for SCZ or T2D in genome wide association study (GWAS) as the basis and retrieved their nearest interactive partners from human protein interaction data to construct a protein-protein interaction network. Next, we selected those novel candidate genes from the network that interact with both SCZ related proteins and T2D related proteins. In this way, we prioritized a set of new candidate genes related to both diseases. Moreover, considering that different biological processes for these two diseases may share the same susceptibility genes, we conducted pathway enrichment analysis with those susceptibility genes related to two diseases, and identified the pathways common to these two diseases and those genes participating into those pathways. Through the pathway analysis, we tried to link the pathogenetic association between the two diseases at the molecular level.

## Materials and methods

### Susceptibility gene sets of SCZ and T2D

**Figure 1:**
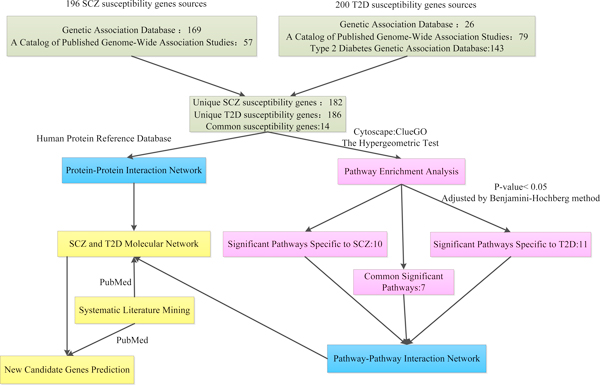
**A detailed flow chart for the analysis process**. The green, pink, blue and yellow denote gene sources, pathway enrichment analysis, network construction and new candidate gene prediction, respectively.

SCZ susceptibility genes were extracted from two publicly available databases: Genetic Association Database [18,19] and A Catalog of Published Genome-Wide Association Studies [20,21]. The former is an archive of human genetic association studies of complex diseases and disorders, which includes summary data extracted from published papers in peer reviewed journals on candidate gene and GWAS studies (updated to June 9, 2012); the latter is an online catalogue of SNP-trait associations from published genome-wide association studies for use in investigating genomic characteristics of trait/disease-associated SNPs (TASs) (updated by June 22, 2012). T2D susceptibility genes were collected from three main sources: the first was Type 2 Diabetes Genetic Association Database [22,23], and this database provides specialized information on the genetic risk factors involved in the development of T2D. Among the data in this database, we only selected genes reported in more than two independent studies. The other two data sources were the same as SCZ genes. The follow-up analyses are based on these two susceptibility genes sets. A detailed flow chart of my methodology is illustrated in Figure 1.

### Significant pathway enrichment analysis

To carry out the pathway enrichment analysis, we uploaded SCZ and T2D susceptibility genes into Cytoscape as cluster 1 and cluster 2, respectively, and ClueGO was used for pathway enrichment analysis for all those genes [24]. Two pathway databases, Kyoto Encyclopedia of Genes and Genomes (KEGG) pathway [25,26] and BioCarta pathway [27,28], were selected for pathway enrichment analysis. Those susceptibility genes were mapped to their enriched pathways based on the hypergeometric test, and p-value was corrected by Benjamini-Hochberg method [29]. It is possible that genes from both clusters are associated with one pathway, but in different proportions. Here we defined an enriched pathway specific to one of the clusters if over 66% genes in the pathway are from this cluster. Pathways with adjusted p-value < 0.05 were regarded as significant enriched pathways and were selected for further analysis.

### Pathway-pathway interaction network construction

To visually represent relationships between the selected significant pathways, a pathway-pathway interaction network was created, in which the node represented the significant pathway, the edge between the significant pathways was defined according to kappa scores which were calculated based on any pathway pair shared genes in a similar way as described by DAVID software [30]. The different proportion of the genes from the analyzed clusters was represented with a colour gradient from blue for the first cluster genes, to red for the second cluster. Approximately equal proportions of the two clusters were represented in light-yellow. The genes shared by any pathway pair and those mapped to corresponding significant pathways were also displayed in this network as small nodes with different colours to distinguish them from pathway nodes. The network was automatically laid out using the Organic layout algorithm supported by Cytoscape.

### Protein-protein interaction data

Protein-Protein interaction data was downloaded from Human Protein Reference Database (HPRD, version: 13 April, 2010) [31,32]. After removing self interactions and disperse nodes, we ended up with 36,727 interactions which cover 9,205 human genes. All proteins encoded by unique susceptibility genes of two diseases were mapped into HPRD, and then we extracted those proteins that directly interact with our susceptibility proteins, and constructed a protein-protein interaction network in which a node is a protein and an edge represents interaction between two proteins.

### New candidate genes prediction

Among all the nearest interacting proteins, those simultaneously interacting with both SCZ and T2D susceptibility gene products were selected, then we constructed a sub-network with them and their interacted susceptibility proteins. Next, we performed extensively literature mining in PubMed to determine whether the relationship between a candidate protein and SCZ or T2D has been supported by previous studies. Based on these two aspects evidence we predicted those genes with pleiotropic effects as the risk factors that may contribute to the pathogenetic association between SCZ and T2D.

## Results

### SCZ and T2D susceptibility gene sets

All the susceptibility genes were selected based on the Genome-Wide Association Studies (GWAS). For SCZ susceptibility genes, we retrieved 169 genes from Genetic Association Database and 57 genes from database of A Catalog of Published Genome-Wide Association Studies. For T2D related genes, we extracted 26 genes and 79 genes from each of above databases, respectively. In addition, we collected 143 genes from Type 2 Diabetes Genetic Association Database. After removing redundancy, we obtained 196 susceptibility genes for SCZ and 200 for T2D, among them, 14 genes are in common for both diseases (Additional file 1).

### Enrichment pathway analysis

To perform functional enrichment tests of the susceptibility genes, we uploaded SCZ and T2D related genes, named as cluster 1 and cluster 2 respectively, into ClueGO, a Cytoscape plug-in to decipher biological networks, and mapped them to their enrichment pathways. Here, considering the incomplete of each pathway annotation system, we selected two main pathway databases, KEGG and BioCarta to conduct our analysis. As a result, we ended up with 10 significant pathways (with adjusted p-value < 0.05) specific to SCZ, 11 significant pathways specific to T2D, and 7 pathways for both diseases (Table 1). Here we defined an enriched pathway specific to one of the clusters if over 66% genes (the default value in the system) in the pathway are from this cluster. Interestingly, some of the enriched pathways, even though they were classified as one of the clusters based on the statistics, they included genes for both SCZ and T2D, such as Adipocytokine signaling pathway and PPAR signaling pathway, both of them were clustered as T2D pathways. In fact, for 18 susceptibility genes in the Adipocytokine signaling pathway, 4 of them are related to SCZ, while 12 of them are identified to T2D related genes, and the rest 2 genes have been linked to both SCZ and T2D. PPAR signaling pathway includes 13 T2D related genes and 2 SCZ related genes. Neuroactive ligand-receptor interaction pathway and Calcium signaling pathway [33] were enriched as SCZ pathways. There are 35 genes in Neuroactive ligand-receptor interaction pathway, and 26 of them are related to SCZ, while the rest 9 genes come from T2D gene list. Calcium signaling pathway contains 18 genes implicated to SCZ, and 5 genes linked to T2D.

**Table 1:** The 28 significant pathways analysed using ClueGO.

Significant Pathways	Specific To cluster	Adjust P-Value	Total number	Gene source		
				
				unique SCZ gene count	unique T2D gene count	Common gene count
Adipocytokine signaling pathway	T2D	3.91E-09	18	4	12	2
Neuroactive ligand-receptor interaction	SCZ	4.01E-09	35	26	9	0
Maturity onset diabetes of the young	T2D	1.94E-08	11	0	11	0
Type II diabetes mellitus	T2D	1.98E-08	14	1	12	1
PPAR signaling pathway	T2D	9.33E-07	15	2	13	0
Calcium signaling pathway	SCZ	5.03E-06	23	18	5	0
Visceral Fat Deposits and the Metabolic Syndrome	T2D	6.57E-05	5	0	4	1
Type I diabetes mellitus	SCZ	7.12E-05	10	8	1	1
Corticosteroids and cardioprotection	BOTH	8.96E-05	6	3	2	1
Low-density lipoprotein (LDL) pathway during atherogenesis	T2D	3.28E-04	4	0	4	0
Insulin signaling pathway	T2D	9.20E-04	16	1	15	0
Graft-versus-host disease	SCZ	0.001587097	8	6	1	1
IL-10 Anti-inflammatory Signaling Pathway	BOTH	0.001671861	4	1	1	2
Basic mechanism of action of PPARa, PPARb(d) and PPARg and effects on gene expression	T2D	0.001780493	3	0	3	0
Actions of Nitric Oxide in the Heart	BOTH	0.002369651	5	2	3	0
Erythropoietin mediated neuroprotection through NF-kB	BOTH	0.003426765	4	2	1	1
Allograft rejection	SCZ	0.003509	7	5	0	2
g-Secretase mediated ErbB4 Signaling Pathway	SCZ	0.005740212	3	3	0	0
Msp/Ron Receptor Signaling Pathway	BOTH	0.005740212	3	1	1	1
Autoimmune thyroid disease	SCZ	0.020029548	7	6	0	1
Alzheimer's disease	BOTH	0.020096651	5	1	2	2
Free Radical Induced Apoptosis	SCZ	0.020961818	3	2	0	1
Role of PPAR-gamma Coactivators in Obesity and Thermogenesis	T2D	0.020961818	3	0	3	0
Asthma	SCZ	0.023911463	5	3	0	2
AKT Signaling Pathway	SCZ	0.024540904	4	3	1	0
Hematopoietic cell lineage	BOTH	0.027660527	9	4	4	1
Regulation of PGC-1a	T2D	0.029865431	3	1	2	0
Role of ERBB2 in Signal Transduction and Oncology	T2D	0.030339933	4	1	3	0

SCZ: schizophrenia; T2D: type 2 diabetes mellitus. The p-value is adjusted by Benjamini-Hochberg method.

**Figure 2:**
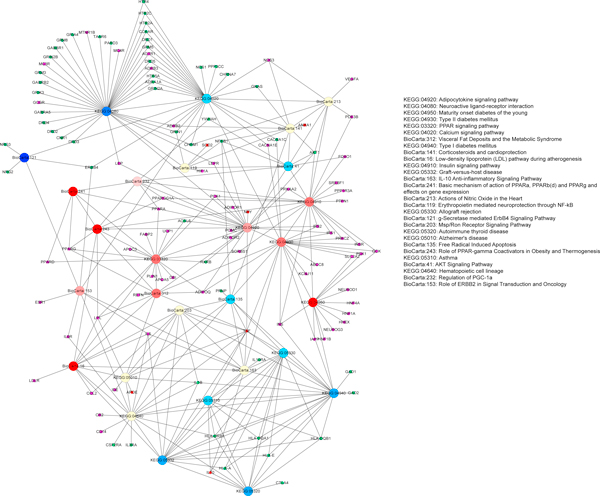
**Pathway-pathway interaction network**. The large node represents pathway, blue for SCZ, red for T2D, light-yellow for both. The small node represents gene mapped to corresponding pathways. Small nodes in green and purple are genes from SCZ and T2D susceptibility gene lists, respectively. The edge represents crosstalk between any two pathways. The network is automatically laid out using the Organic layout algorithm supported by Cytoscape.

Next, to explore the association and crosstalk between those different enriched pathways, we constructed a pathway-based network with all those 28 significant pathways in which a large node is a pathway and an edge represents crosstalk between two pathways through their shared genes (Figure 2). The genes shared by any pathway pair and those mapped to corresponding significant pathways were displayed in this network as small nodes with different colours to distinguish them from pathway nodes. From the pathway-pathway interaction network, it can be observed that many genes are shared by multiple pathways, such as TNF shared by over 12 different signaling pathways, AKT1 participating into 4 different signalling pathways (Additional file 2).

### New candidate risk gene inference

To infer new genes associated with both SCZ and T2D, we conducted network analysis based on protein-protein interaction (PPI). First, we downloaded human PPI data from HPRD. Next, we mapped all 382 unique SCZ and T2D susceptibility gene related proteins (susceptibility proteins) to the human PPI data, only proteins that have their interacting partners in the HPRD data were selected in our further analysis. Then we retrieved those susceptibility proteins with their nearest interacting neighbours from the PPI data. After removing self-interaction and duplicates, the final network included a total of 2,104 nodes and 3,155 interactions (Additional file 3). Those 2,104 proteins included 143 SCZ susceptibility proteins, 138 T2D susceptibility proteins, 12 common susceptibility proteins and 1,811 their direct interaction partners. Among the 1,811 protein partners, there were 1,108 proteins that interact with more than one SCZ susceptibility proteins, 1,067 proteins with more than one T2D susceptibility proteins, and 364 proteins with both diseases' susceptibility proteins. We proposed those 364 proteins as new candidate risk factors for both SCZ and T2D according to function association (guilt by association) rule. Function association refers to that if two proteins interact with one another, they usually participate in the same, or related, cellular functions. Based on this assumption, new functions of proteins can be inferred with their interaction partners.

**Figure 3:**
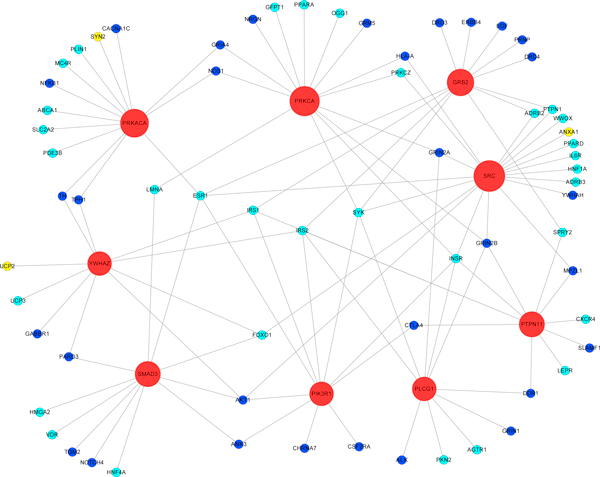
**The sub-network of 9 hub proteins and their interacted susceptibility proteins**. Nodes in blue represent SCZ susceptibility proteins; nodes in light-green represent T2D susceptibility proteins; nodes in yellow represent SCZ and T2D common susceptibility proteins. Large nodes in red are 9 hub proteins. The size of hub proteins reflects the degree in the network.

The 364 candidate proteins and their interacted susceptibility proteins may provide new relationship for elucidating the common molecular pathways that may underlie both SCZ and T2D. So we extracted those 364 candidate proteins and their interacted susceptibility proteins from the entire network to construct a sub-network (Additional file 4). In this sub-network, among all 364 candidate proteins, 9 proteins closely interacted with both multiple SCZ and T2D susceptibility proteins (with both interacting partners ≥ 5) and were regarded as hub proteins, these hub proteins include SRC, PRKACA, PRKCA, GRB2, PTPN11, SMAD3, YWHAZ, PIK3R1 and PLCG1 (Figure 3). Some of these hub proteins are the components of our identified enriched pathways (Table 2).

**Table 2:** Six hub proteins that involved in previous enriched pathways.

Previous enriched pathways	Adjusted P-Value	Hub proteins
Calcium signaling pathway	7.42E-06	PLCG1 PRKACA PRKCA
g-Secretase mediated ErbB4 Signaling Pathway	4.33E-03	PRKCA
Adipocytokine signaling pathway	6.41E-08	PTPN11
Insulin signaling pathway	5.46E-06	GRB2 PIK3R1 PRKACA
AKT Signaling Pathway	4.26E-04	PIK3R1
Type II diabetes mellitus	6.67E-03	PRKACA PIK3R1

To verify whether the function association approach is reasonable to infer the function relationships of those proteins to the two diseases, we performed systematic literature mining to survey whether those candidate genes are reported in PubMed articles for SCZ and T2D. As a result, we found that 59 candidate genes have been connected to SCZ [34-38], 77 candidate genes have been linked to T2D [39-43], while 25 candidate genes [44-49] have been implicated to both SCZ and T2D with various studies (Additional file 5). Totally, 161 candidate genes (~45% of all candidate genes) have been related to either SCZ or T2D or both diseases with various experimental approaches, further proving the rationale of "function association" in the application of disease related gene inference. We proposed that genes encoding those 33 proteins (9 hub proteins and 25 proteins, with one common protein) could be high-priority candidate genes contributing to pathogenetic association between SCZ and T2D.

### SCZ and T2D molecular network construction

**Figure 4:**
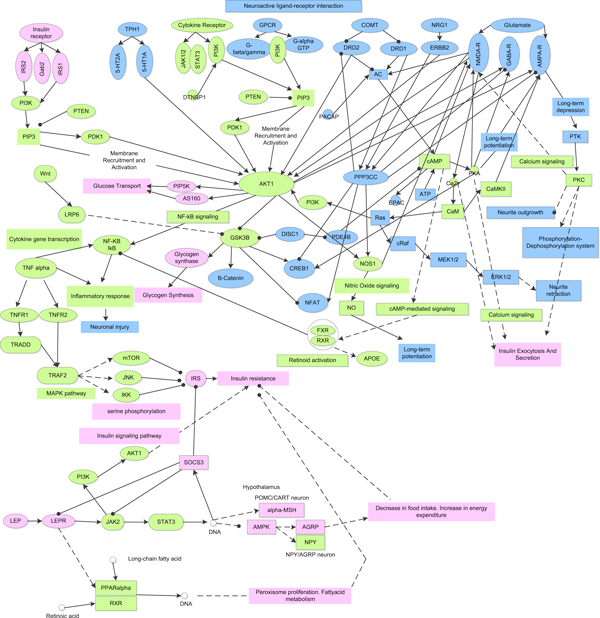
**The SCZ & T2D molecular network**. The potential underlying common molecular mechanism between SCZ and T2D. Nodes in blue, pink and green background are related to SCZ, T2D and common progression, respectively. Solid and dashed lines represent direct and indirect regulation; lines with arrow and spot represent activation and inhibition, respectively.

Last, to explore the potential relationships of those identified genes and two diseases, based on our constructed pathway network, protein-protein interaction and literature survey, we developed a SCZ & T2D molecular network (STMN), in which the relationships between those susceptibility genes/proteins and the two diseases have been inferred (Figure 4).

## Discussion

As complex diseases, both SCZ and T2D have attracted more and more attentions in the research communities for their significant increasing prevalence during past decades. Clinical studied have reported that the risk of T2D is increased in schizophrenic patients and T2D is one of the leading causes of morbidity and mortality in individuals affected with SCZ-related disorders (i.e., SCZ, schizoaffective disorder, and schizophreniform disorder) [50,51]. There have been numerous reports of susceptibility genes or loci to SCZ or T2D, however, few genes have been confirmed to link to the two diseases and the mechanisms for the association remain unclear. The limited success in detection of genetic factors for both diseases has indicated that the diseases are not caused by the dysfunction of a specific molecule or pathway, most likely both diseases are caused by the altered function or expression of many genes, which may individually contribute to only a small risk, but their collective dysfunctional effects interfere with the function of several biological pathways that eventually produce the clinical outcome [52]. Therefore, studies based on network and pathway interaction naturally are the choice for both of the diseases and their association. To our knowledge, our study is the first network and pathway-based systematic analyses for the pathogenetic association between SCZ and T2D by using susceptibility genes generated from various researches. For many complex diseases, including SCZ and T2D, there are no applicable gene signatures in clinical to detect them in early stages. The new discovered common susceptible genes related to the pathogenetic association between SCZ and T2D could be potentially used as candidates to signify the co-occurrence of SCZ with T2D.

From our enrichment pathway analysis results and the pathway-pathway interaction network, we observed that many genes are shared by several pathways, such as TNF shared by 12 enriched pathways and AKT1 shared by 4 enriched pathways. Those genes that participate in several pathways could be the key components for the pathway crosstalks and the potential risk factors for the SCZ and T2D association.

As a serine/threonine kinase, AKT is a key regulator of many signal transduction processes mediated by protein phosphorylation and a central molecule in regulating multiple cellular processes such as glucose metabolism, transcription, apoptosis, cell proliferation, angiogenesis, and cell motility [53]. AKT is activated by phosphoinositide 3-kinase (PI3K), which itself is activated by several upstream signaling pathways, Neuroactive ligand-receptor interaction pathway is the major one for the activation of PI3K. Through PI3K, AKT is regulated by many proteins, such as insulin receptors, receptor tyrosine kinases, G protein coupled receptors, cytokine receptors, etc., then controls diverse biological responses such as programmed cell death, cell proliferation, migration, and metabolic processes. Recently, accumulating evidences suggest that impaired AKT signaling plays a role in the pathogenesis of SCZ [54]. The potential molecular mechanisms underlying the role of AKT signaling in SCZ has contributed to the AKT dysfunction. Activated AKT can phosphorylate a number of other molecules, one of them is the strong clinically relevant target, glycogen synthase kinase-3 (GSK3) [53]. GSK3 has been confirmed to play several roles in glucose metabolism, differentiation and development, intracellular trafficking, apoptosis, and regulation of gene transcription [55]. In the brain, both GSK3 and AKT have been proposed to modulate synaptic plasticity [56]. AKT1 activation has been reported to be reduced in the hippocampus and frontal cortex of SCZ patients compared with healthy controls [54]. Other studies have further provided the evidence of a reduction of AKT1 mRNA and protein levels in peripheral blood, prefrontal cortex, and hippocampus in SCZ patients [57,58]. Moreover, the single SNP that is associated with reduced expression of AKT1 in peripheral lymphocytes is associated with brain volume reductions in caudate and right prefrontal cortex [59].

The AKT signaling pathway also plays a pivotal role in the metabolic functions of insulin in the liver. AKT regulates glycogenesis through the phosphorylation of GSK3, GSK3 phosphorylates glycogen synthase and converts it to the less-active (glucose-6-phosphate dependent) form, thus inhibits glycogen synthesis. In contrast to the phosphorylation of AKT for its activation [53], constitutively activated GSK3 in resting cells requires phosphorylation by kinases such as AKT to inactivate it [60]. Interestingly, 68% less expression of AKT1 has been detected in the lymphocytes of SCZ patients compared with healthy controls [54]. Significant reduction of AKT1 expression and deregulation of AKT1-associated pathways have recently also been reported in peripheral blood cells of schizophrenia patients [61]. The impaired activation of AKT in SCZ patients could result in the higher activity of GSK3 in blood, which eventually causes the reduction of glycogen and inhibition of glucose with the increase of blood glucose levels. In addition, AKT1 has also been associated with other signaling pathways, such Dopamine pathways, Wnt signalling pathway and Adipocytokine signaling pathway. The dysfunction of these signaling pathways with impaired AKT1 all has significant impact on the SCZ or T2D, which is consistent with our analysis result. Taken together, AKT signaling pathway could be one of the pivotal pathways to bridge the association between SCZ and T2D, AKT1 gene, together with GSK3 gene in this pathway, may be responsible for the co-occurrence of SCZ and T2D.

Leptin (LEP) gene (Figure 2) is involved in the pathways of Neuroactive ligand-receptor interaction and Adipocytokine signaling in our pathway-pathway interaction network. Leptin is secreted by adipose tissue and signifies the endocrine function of adipose tissue. An increase in leptin signals can affect the neuronal targets in the hypothalamus. Leptin activates Janus-activating kinase2 (Jak2) and STAT3, leading to activate alpha-MSH and CART in POMC/CART neuron, and inhibit NPY and AGRP in NPY/AGRP neuron. The Neuroactive ligand-receptor interaction pathway contains G protein-coupled receptors (GPCRs) of dopamine and serotonin which have been proposed to play an important role in the pathophysiology of SCZ. Previous studies have suggested that LEP may associate with SCZ [62,63]. Adipocytokine signaling pathway has been specifically linked to T2D. As a component for Adipocytokine signalling pathway, LEP is considered to be an important regulator in the pathophysiology of T2D diseases. In our constructed STMN, we also observed a crosstalk between leptin and insulin in the hypothalamus. In addition, leptin can activate AKT1 through the activation of PI3K, and possibly through JAK2, thus providing a mechanism for regulation of target genes, the same as in Insulin signaling pathway. Therefore, the crosstalk between above two pathways also implies the underlying pathogenetic association between SCZ and T2D.

Corticosteroids and cardioprotection pathway, a pathway both for SCZ and T2D, was reported to be associated with SCZ [64,65] and T2D [66]. It interlinks to Calcium signaling pathway and Insulin signaling pathway. Interestingly, the crosstalk between Corticosteroids and cardioprotection pathway and Insulin signaling pathway is mediated by AKT according to our pathway-based network. Previous study also has shown that Calcium signaling pathway is associated with dopamine-induced cortical neuron apoptosis which is considered as an important mechanism in SCZ pathogenesis [67]. Meanwhile, Actions of Nitric Oxide in the Heart, another pathway for both SCZ and T2D, is a crosstalk between Calcium signaling pathway and Insulin signaling pathway either. Previous study indicated that Nitric oxide was involved in pathophysiology of SCZ [68]. IL-10 Anti-inflammatory signaling pathway is an immune-related pathway. Accumulated evidence from epidemiological, clinical and animal studies suggests that immune-related pathway may play a key role in the development of mental diseases including SCZ and mood disorders [69,70]. IL-10 Anti-inflammatory Signaling Pathway has been reported previously to be involved in pathophysiology of SCZ [71] and T2D [72], respectively. Therefore, the above evidence suggests that IL-10 Anti-inflammatory signaling pathway may be involved in the pathogenetic association between SCZ and T2D. In another perspective, due to inflammation contributes to injury or enhances CNS vulnerability, and acute inflammation can also be shifted to a chronic inflammatory state and adversely affect brain development, therefore, through efficient anti-inflammatory and reparative processes, inflammation may resolve without any harmful effects on the brain. Alternatively, intervention of TNF-α, before the progressive loss of beta cell function, may yield promising results in the treatment of T2D. Since IL-10 is a cytokine with potent anti-inflammatory properties, it represses the expression of inflammatory cytokines such as TNF-α, IL-6 and IL-1 by activated macrophages. The anti-inflammatory actions of IL-10 may be therapeutically useful by intervention of TNF-α, IL-1 or IL-6 to avoid inflammatory response, then to decrease the CNS vulnerability, further to reduce the chance to trigger T2D.

In our inferred new candidate risk factors, 9 proteins interact with multiple proteins involved in both diseases with high connectivity, 6 of them are found to be the components of our enriched pathways (Table 2). Among them, PRKACA is shared by Type II diabetes mellitus, Insulin signaling pathway and Calcium signaling pathways; PIK3R1 is a common molecule of AKT signaling, Insulin signaling and Type II diabetes mellitus pathways; PRKCA is a component for both of Calcium signaling and g-Secretase mediated ErbB4 signaling pathways while PLCG1 for Calcium signaling pathway, PTPN11 for Adipocytokine signaling pathway and GRB2 for Insulin signaling pathway. All of those proteins may be associated with both SCZ and T2D through participating into related signaling pathways and interacting with other disease related susceptibility genes, then further enhancing the linkage between SCZ and T2D.

For the rest of three hub proteins, SRC, SMAD3 and YWHAZ, they may also play some role in contributing to pathogenic association between SCZ and T2D. Src is a tyrosine kinase. In the sub-network, it interacts with 7 and 13 SCZ and T2D related proteins, respectively. Src has been associated with SCZ, the potential molecular mechanism is that the NRG1-ErbB4 pathway, which is a candidate pathway participated in cognitive dysfunction in SCZ, affects NMDAR hypofunction through modulation of Src activity. In mouse model, NRG1-ErbB4 signaling blocks Src enhancement of NMDAR-mediated synaptic currents [73]. Although there has no report about Src implicated with T2D, from the sub-network, we observed that Src links to multiple T2D related proteins, such as INSR, an insulin receptor, and AKT1. Given that the Src protein is a tyrosine kinase, which plays critical roles in the activiation of multiple signaling pathways [74], we speculate that SRC is a potential candidate gene with pleiotropic effects that affects both SCZ and T2D.

SMAD3 is a member of SMAD protein family that are signal transducers and transcriptional modulators that mediate multiple signaling pathways. One of those signaling pathway is the transforming growth factor beta (TGF-β) pathway [75], TGF-β plays an important role in regulation of insulin gene transcription and β-cell function [39], it is also a key mediator in the development of diabetic complications. TGF-β exerts its biological effects by activating downstream mediators, called Smad2 and Smad3. Recent studies have demonstrated that under disease conditions Smad3 act as signal integrators and interact with other signaling pathways, such as the MAPK and NF-κB pathways [76]. In adult Smad3 null mice, TGF-β signaling through Smad3 is needed to maintain the rate of cell division of neuronal precursors in the adult brain and hence the amount of neurogenesis [77]. Another Smad family member - Smad4 has been proven to be related to SCZ, since forebrain-specific Smad4 knock-out mice shows typical endophenotype of schizophrenia [78]. Taken together, these data add new evidence to support our hypothesis that the Smad3 may link to both SCZ and T2D by interacting with multiple signaling pathways as a signal integrator.

YWHAZ gene product belongs to the 14-3-3 family of proteins which mediate signal transduction by binding to phosphoserine-containing proteins. The encoded protein interacts with IRS1 protein, and is a negative regulator for insulin signal transduction, suggesting its role in regulating insulin sensitivity [79]. Previous study has also indicated that the YWHAZ gene is a potential risk factor for paranoid SCZ, although the potential mechanism of how this gene affected biological functions in the brain is unknown [80]. Therefore, our hypothesis tentatively assumes that the YWHAZ may also be a pleiotropic gene, which participates in the pathogenetic linkage between SCZ and T2D diseases.

For the rest of new candidate genes, although the number of interaction partners for them is various and less than those hub proteins in the PPI network, 25 of them, including well known genes, TP53, GSK3 and RXRA, are still supportedly associated with SCZ and T2D by text mining. Various data have indicated that they all have been implicated in both of the diseases (Additional file 5). For those genes without literature support, they may also be involved in differential but intertwined SCZ and T2D pathogenetic processes. Further experiments need to carry out to verify those associations.

The new candidate genes are inferred from the PPI, however, it is worth pointing out that the PPI we used in the study represents a static relationship between each protein pair. In real biological processes, such as pathogenetic conditions or different development stages, gene expression has spatiotemporal pattern, the same as protein-protein interaction. Therefore, different implicated genes may participate into SCZ and T2D diseases in different stages and play different roles in the association with the SCZ and T2D. By integrating multiple dimensional data, it can be expected that network-based approach, combined with other multiple resources, will provide great help to decipher the coordination and functional roles of those implicated genes in complex diseases. Furthermore, it is well known that many proteins in signaling pathways are drug targets. Our pathway-based network has revealed that many susceptible genes linking SCZ and T2D participate into different signaling pathways and have pleiotropic effects, their encoded proteins could be good candidates as drug targets to treat this complex disease, and selectively targeting those dysfunctional proteins in different signaling pathways with synergetic effect could potentially have better treatment outcome.

There are certain limitations in our study. First, those prioritized SCZ genes and T2D related genes we used are all from GWAS. Considering the inherent drawbacks of GWAS approach with its noise and high false positive rate, some of the genes may not be truly associated with both of the diseases, which will certainly affect the pathway enrichment analysis result and our inference of new candidate risk genes for the association of SCZ and T2D. Second, the incomplete pathway annotation systems for each pathway database could also negatively contribute to the pathway network construction and the pathway crosstalk interpretation. Nevertheless, our results still present novel and promising explanation for the association between SCZ and T2D, these novel relationships could offer new insights into these two diseases' etiology.

## Conclusions

We have successfully built the pathogenetic association between SCZ and T2D based on their enriched pathway crosstalk. Through the integration of multiple level analysis results, including pathway crosstalk, PPI and literature survey, we revealed some potential molecular mechanisms and multiple susceptibility genes that could exert pleiotropic effects shared by two diseases. Totally 364 candidate proteins that directly interacted with both our SCZ and T2D susceptibility proteins have been identified, 33 of them have been prioritized as high significant genes linking to both of SCZ and T2D. Although there are certain limitations for our analysis processes, our preliminary findings can provide an alternative direction for other researchers to explore the linkage between these two diseases.

Currently, many chromosomal intergenic regions and SNPs on human genome have been associated with diseases. However, no gene has been identified in those regions or to host those SNPs. It can be anticipated that with the emergence and significant progress of new technologies, such as next generation sequence technology [81,82], more and more genes and transcribed regions will be discovered in human genome [83,84] and those unrealized expression genes in the current intergenic regions will be indentified and linked to the diseases. Those will definitely facilitate the investigation of those complex diseases, and help us to reshape the potential underlying genetic mechanisms for those complex diseases.

## Competing interests

The authors declare that they have no competing interests.

## Authors' contributions

TS and ZY conceived and designed the study. YL, ZL, MZ performed analyses. TS, ZY, YL, ZL and YD wrote the manuscript, TS finalized the manuscript.

## Supplementary Material

Additional file 1 **196 SCZ and 200 T2D susceptibility gene sets**. Click here for file

Additional file 2 **Pathway shared genes and their involved pathways**. Click here for file

Additional file 3 **Protein-Protein Interaction Network**. This network consists of 2,104 nodes and 3,155 edges, nodes represent proteins, node size stands for its degree, edges represent interaction between two proteins. Nodes in blue are 143 SCZ susceptibility proteins; nodes in green are 138 T2D susceptibility proteins; nodes in yellow are common susceptibility proteins; remainder nodes in purple are 1,811 candidate proteins. Click here for file

Additional file 4 **Sub-network extracted from **Additional file 3. This network consists of 580 nodes and 1,266 edges, node attributes refer to Additional file 3. Click here for file

Additional file 5 **Literature mining results for the 364 new candidate genes**. 364 candidate genes are in the first column; the second column hosts the number of interactions from SCZ susceptibility proteins; the third column lists the PubMed ID for the reported protein associated with SCZ; the fourth column is the interactions of SCZ. The corresponding results of T2D are displayed in the following columns. Genes in pink background are 25 candidate genes which have been implicated in both SCZ and T2D with various studies. Click here for file
