# Supplementary material for: Exploring the pathogenetic association between schizophrenia and type 2 diabetes mellitus diseases based on pathway analysis
Source: BMC Med Genomics. 2013 Jan 23;6(Suppl 1):S17. doi: 10.1186/1755-8794-6-S1-S17 (PMC3552677; doi:10.1186/1755-8794-6-S1-S17)
Supplement: Additional file 2 — Pathway shared genes and their involved pathways. [file 1755-8794-6-S1-S17-S2.docx]

| Pathways shared genes | Involved pathways |
| --- | --- |
| TNF | Free Radical Induced Apoptosis  Adipocytokine signaling pathway  Type II diabetes mellitus  Visceral Fat Deposits and the Metabolic Syndrome  Alzheimer's disease  IL-10 Anti-inflammatory Signaling Pathway  Msp/Ron Receptor Signaling Pathway  Hematopoietic cell lineage  Type I diabetes mellitus  Asthma  Allograft rejection  Graft-versus-host disease |
| HLA-DRB1 | Hematopoietic cell lineage  Type I diabetes mellitus  Asthma  Autoimmune thyroid disease  Allograft rejection  Graft-versus-host disease |
| AKT1 | Actions of Nitric Oxide in the Heart  Corticosteroids and cardioprotection  AKT Signaling Pathway  Adipocytokine signaling pathway  Insulin signaling pathway |
| HLA-DQA1 | Type I diabetes mellitus  Asthma  Autoimmune thyroid disease  Allograft rejection  Graft-versus-host disease |
| IL6 | Role of ERBB2 in Signal Transduction and Oncology  Low-density lipoprotein (LDL) pathway during atherogenesis  IL-10 Anti-inflammatory Signaling Pathway  Hematopoietic cell lineage  Graft-versus-host disease |
| HLA-DQB1 | Type I diabetes mellitus  Asthma  Autoimmune thyroid disease  Allograft rejection  Graft-versus-host disease |
| NFKB1 | Erythropoietin mediated neuroprotection through NF-kB  Free Radical Induced Apoptosis  Corticosteroids and cardioprotection  AKT Signaling Pathway  Adipocytokine signaling pathway |
| IL1B | Alzheimer's disease  Msp/Ron Receptor Signaling Pathway  Hematopoietic cell lineage  Type I diabetes mellitus  Graft-versus-host disease |
| LPL | Low-density lipoprotein (LDL) pathway during atherogenesis  Role of PPAR-gamma Coactivators in Obesity and Thermogenesis  PPAR signaling pathway  Visceral Fat Deposits and the Metabolic Syndrome  Alzheimer's disease |
| PPARA | \| Regulation of PGC-1a \| \| --- \| \| Basic mechanism of action of PPARa, PPARb(d) and PPARg and effects on gene expression \| \| PPAR signaling pathway \| \| Adipocytokine signaling pathway \| |
| IL10 | \| IL-10 Anti-inflammatory Signaling Pathway \| \| --- \| \| Asthma \| \| Autoimmune thyroid disease \| \| Allograft rejection \| |
| PPARGC1A | \| Regulation of PGC-1a \| \| --- \| \| Role of PPAR-gamma Coactivators in Obesity and Thermogenesis \| \| Adipocytokine signaling pathway \| \| Insulin signaling pathway \| |
| INS | \| Insulin signaling pathway \| \| --- \| \| Type II diabetes mellitus \| \| Maturity onset diabetes of the young \| \| Type I diabetes mellitus \| |
| HLA-A | \| Type I diabetes mellitus \| \| --- \| \| Autoimmune thyroid disease \| \| Allograft rejection \| \| Graft-versus-host disease \| |
| ERBB4 | \| g-Secretase mediated ErbB4 Signaling Pathway \| \| --- \| \| Role of ERBB2 in Signal Transduction and Oncology \| \| Calcium signaling pathway \| |
| GRIN1 | \| Erythropoietin mediated neuroprotection through NF-kB \| \| --- \| \| Calcium signaling pathway \| \| Neuroactive ligand-receptor interaction \| |
| IRS2 | \| Adipocytokine signaling pathway \| \| --- \| \| Insulin signaling pathway \| \| Type II diabetes mellitus \| |
| CHRM1 | \| Actions of Nitric Oxide in the Heart \| \| --- \| \| Calcium signaling pathway \| \| Neuroactive ligand-receptor interaction \| |
| GCK | \| Insulin signaling pathway \| \| --- \| \| Type II diabetes mellitus \| \| Maturity onset diabetes of the young \| |
| PCK1 | \| PPAR signaling pathway \| \| --- \| \| Adipocytokine signaling pathway \| \| Insulin signaling pathway \| |
| IRS1 | \| Adipocytokine signaling pathway \| \| --- \| \| Insulin signaling pathway \| \| Type II diabetes mellitus \| |
| NOS3 | \| Actions of Nitric Oxide in the Heart \| \| --- \| \| Corticosteroids and cardioprotection \| \| Calcium signaling pathway \| |
| ADRB2 | \| Corticosteroids and cardioprotection \| \| --- \| \| Calcium signaling pathway \| \| Neuroactive ligand-receptor interaction \| |
