# Supplementary figures and images for: Exploring the pathogenetic association between schizophrenia and type 2 diabetes mellitus diseases based on pathway analysis
Source: BMC Med Genomics. 2013 Jan 23;6(Suppl 1):S17. doi: 10.1186/1755-8794-6-S1-S17 (PMC3552677; doi:10.1186/1755-8794-6-S1-S17)

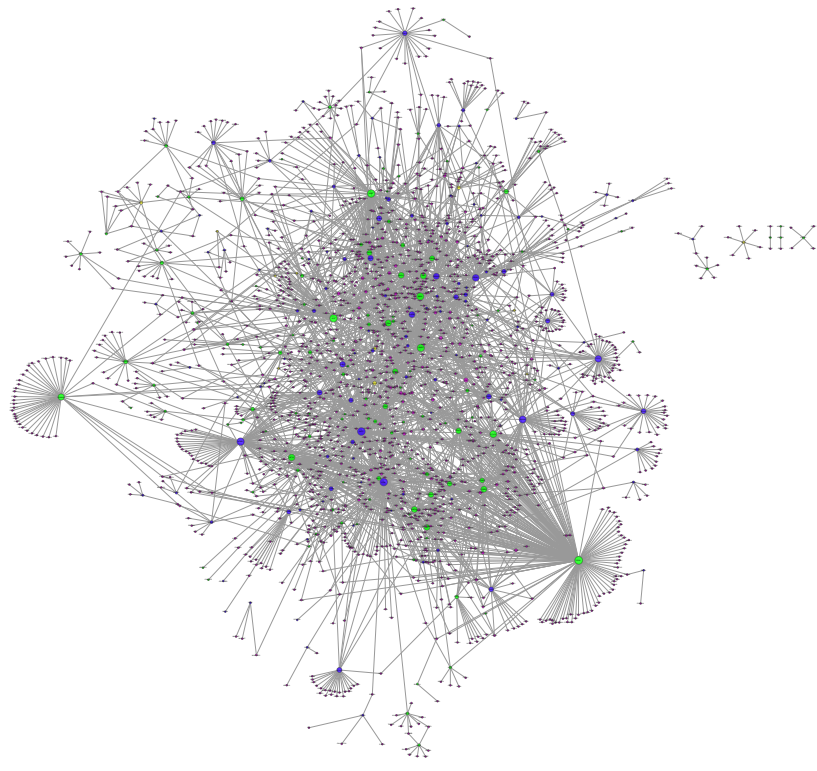

Supplement: Additional file 3 — Protein-Protein Interaction Network. This network consists of 2,104 nodes and 3,155 edges, nodes represent proteins, node size stands for its degree, edges represent interaction between two proteins. Nodes in blue are 143 SCZ susceptibility proteins; nodes in green are 138 T2D susceptibility proteins; nodes in yellow are common susceptibility proteins; remainder nodes in purple are 1,811 candidate proteins. [file 1755-8794-6-S1-S17-S3.pdf]

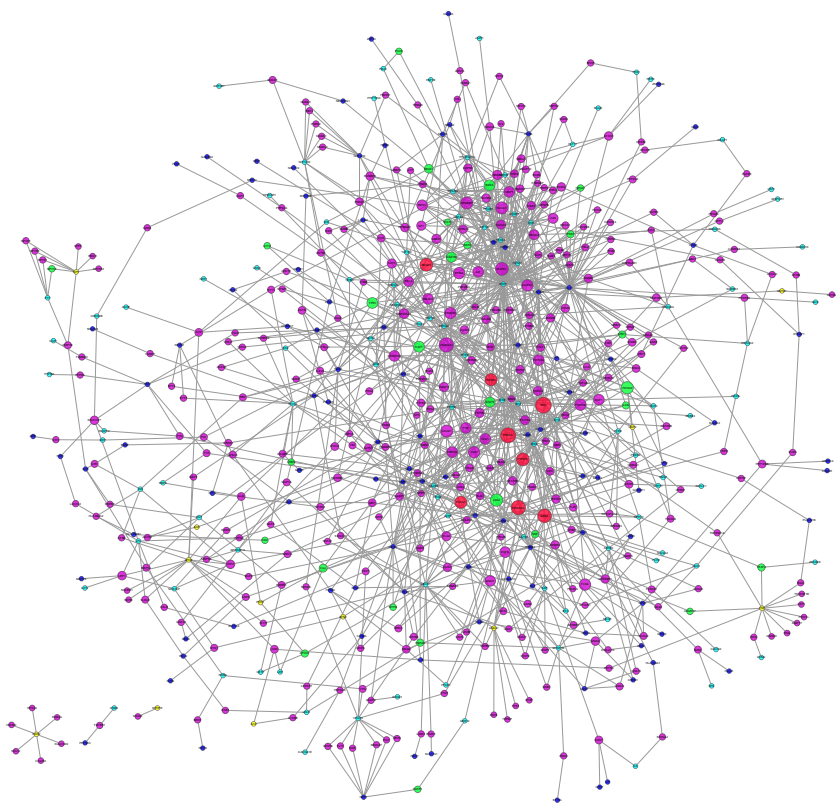

Supplement: Additional file 4 — Sub-network extracted from Additional file 3. This network consists of 580 nodes and 1,266 edges, node attributes refer to Additional file 3. [file 1755-8794-6-S1-S17-S4.pdf]
